# *Corynebacterium pseudotuberculosis* mastitis in Egyptian dairy goats

**DOI:** 10.14202/vetworld.2018.1574-1580

**Published:** 2018-11-13

**Authors:** A. M. Nabih, Hany A. Hussein, Safaa A. El-Wakeel, Khaled A. Abd El-Razik, A. M. Gomaa

**Affiliations:** 1 Department of Mastitis and Neonatal Diseases, Animal Reproduction Research Institute, Agriculture Research Center, Giza, Egypt; 2 Department of Animal Reproduction and AI, Veterinary Research Division, National Research Centre, Dokki, Giza, Egypt; 3 Guangdong Haid Institute of Animal Husbandry and Veterinary (GHIAHV), Guangzhou, Guangdong, China

**Keywords:** bacteriological investigation, caprine, *Corynebacterium pseudotuberculosis*, mastitis, phospholipase D, β-subunit of RNA polymerase

## Abstract

**Background and Aim:**

Mastitis is an important threat facing goat milk industry and is the most common cause of culling. Efficient control of mastitis, based on efficient diagnosis of diseased animals, would improve milk production and reproductive efficiency. In subclinical mastitis (SCM), infected goats demonstrate neither udder symptoms nor abnormal milk. *Corynebacterium pseudotuberculosis* is an infectious causative agent of mastitis, mostly results as an extension of infection from the supramammary lymph node, and causes financial losses in the goat industry. This study aimed to estimate the prevalence of SCM with emphasis on *C. pseudotuberculosis* mastitis in Egyptian dairy goats in the selected farms.

**Materials and Methods:**

A total of 336 half milk samples were collected from 177 dairy goats of various crossbreeds, in mid-to-late lactation period, after clinical examination. All samples were examined bacteriologically, while somatic cell count (SCC) was determined only in 180 half milk samples of the clinically healthy milk samples. The isolated and identified *C. pseudotuberculosis* was examined for evidence of virulence genes (Phospholipase D [*pld*] and β-subunit of RNA polymerase [*rpoB*]) by polymerase chain reaction (PCR).

**Results:**

The prevalence of clinical mastitis was 30.5%, while 69.5% of animals were apparently healthy and secreted milk was normal. Of those 180 clinically healthy half milk samples, 96 milk samples (53.33%) showed SCM as detected by SCC (SCC ≥1,000,000 cells/ml). Coagulase-negative staphylococci were the most prevalent bacteria (41.96%), then *Staphylococcus aureus* (37.5%) and *C. pseudotuberculosis* (7.14%). Molecular diagnosis of virulence genes revealed evidence of *pld* gene in 16 isolates (66.66%), and *rpoB* gene in 6 samples (25%) of the 24 bacteriologically isolated *C. pseudotuberculosis*. Here, we describe, for the 1^st^ time, isolation and identification of *C. pseudotuberculosis* from milk of does suffering from SCM in Egypt.

**Conclusion:**

*C. pseudotuberculosis* must be considered for routine bacteriological examination of milk from dairy goats, particularly herds with a history of caseous lymphadenitis. *Pld* gene-based PCR is more reliable than *rpoB* gene-based ones for the diagnosis of *C. pseudotuberculosis*.

## Introduction

The past two decades have seen intensification in dairy goat production with a significant increase in the number of goats worldwide [1,2], as nutritional qualities of goat milk are to great extent similar to human milk, and less allergenic for human than bovine milk [3].

Mastitis is the most serious disease in dairy goats due to financial losses attributed to its negative impact on milk quantity and components [4,5] and is the main cause of culling for sanitary reasons [6]. Besides, milk fom mastitic udder has public health hazard [7].

In dairy goats, the incidence of clinical mastitis may not exceed 5%, while subclinical mastitis (SCM) is common and about 6 times more than clinical affections [8] and associated with production loss, decreased milk quality, increased replacement cost, and considerable treatment expenses [9,10].

In dairy goats, the problem of SCM is exacerbated as infected goats demonstrate neither udder symptoms nor abnormal milk; hence, the identification of disease is delayed [11]. Thus, SCM must be considered as a serious economic disease [12]. In goats, SCM is mainly caused by *Staphylococcus aureus*, coagulase-negative staphylococci (CNS), *Streptococci agalactiae*, Streptococci Group C, and *Mycoplasma* spp. [13].

*Corynebacterium pseudotuberculosis* is one of the infectious causative agents of mastitis, occasionally encountered in goat and sheep. *C. pseudotuberculosis* mastitis is likely to be an extension of infection from the adjacent lymph node [14]. *C. pseudotuberculosis* mastitis was reported in cattle [15,16]. *C. pseudotuberculosis* is the causative agent of caseous lymphadenitis (CLA) [17], characterized by abscess formation in several organs in small ruminants [18]. CLA is a worldwide distributed disease [19], which causes significant financial losses in goat and sheep industry due to decreased milk production, wasting, low reproductive rates, and condemnation of carcasses due to internal abscesses [20,21]. Although rare, *C. pseudotuberculosis* has a public health hazard. It causes lymphadenitis in human and acquired through close contact with diseased animals [22,23].

Phospholipase D (*Pld*) is the most important virulence factor in *C. pseudotuberculosis* [24]. *Pld* is an exotoxin, induces increased vascular permeability through catalyzing sphingomyelin dissociation, resulting in spread and survival of *C. pseudotuberculosis* in cells, and, consequently, the invasion of the body and transport by phagocytes to regional lymph nodes [25,26].

*Pld* gene detection is used as a diagnostic tool for *C. pseudotuberculosis*. More recently, analysis of partial gene sequences from the β-subunit of RNA polymerase (*rpoB*) has been used for the identification of *Corynebacterium* species than analyses based on 16S rDNA. This method has also been successfully used as a powerful identification tool for mycobacterial species [27]. As well as, many authors propose that it may be used to complement the 16S *rRNA* gene analysis in the phylogenetic studies of *Corynebacterium* and *Mycobacterium* species [21,28].

There are several methods for diagnosis of intra-mammary infection (IMI), of those methods is bacteriological examination of milk [29]. However, bacteriological examination is mostly expensive, time-consuming, and milk culture may yield no bacteria from truly infected glands with very low numbers of pathogens or due to inhibitory effect of residual antimicrobials [30]. Consequently, other diagnostic methods such as indirect measurements of somatic cell count (SCC) with the California mastitis test were developed [13,31]. SCC is commonly used worldwide as an indicator for SCM and to evaluate the efficiency of control programs of mastitis in dairy cattle and buffalo [32]. Unfortunately, interpretation of SCC is difficult in goats, because the relationship of bacterial infections and SCC values is not as simple as in cattle since SCC is significantly affected by several non-infectious factors. Other intrinsic factors such as time and number of lactation, milking time, milking routine, seasonality, and food affect SCC [33,34]. In addition, the apocrine nature of milk secretion in goat results in the presence of cytoplasmic particles or epithelial debris hinders the use of DNA-specific counters mandatory [5].

Recently, molecular diagnosis of pathogens has been introduced. Polymerase chain reaction (PCR) and multiplex PCR have been explored as rapid, sensitive, and reliable approaches for the diagnosis of mastitis-causing pathogens [35-37].

Therefore, the aim of this study was to estimate the SCM prevalence of with emphasis on *C. pseudotuberculosis* mastitis in Egyptian dairy goats in the selected farms.

## Materials and Methods

### Ethical approval

All samples were collected as per standard sample collection procedure without giving any stress or harm to the animals. The present work was approved by the Ethical Committee for Medical Research at the National Research Centre and Animal Care Guidelines of the General Organization for Veterinary Services, Egypt.

### Animals

**Figure-1:**
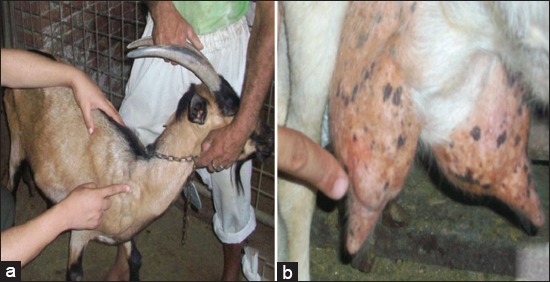
Clinical examination of goats. (a) Case of caseous lymphadenitis infection in prescapular lymph node. (b) Case of abscess in mammary gland with internal palpable abscess.

A total of 177 dairy goats of various crossbreeds located in El Fayoum Governorate, Egypt, were employed in this study. All goats were in mid-to-late lactation at sampling, and some of these animals suffered from CLA with a history of chronicity of infection in these farms (Figure-1). Animals were subjected to clinical examination for the detection of any clinical abnormalities with special attention to the udder by visual inspection and palpation for the detection of clinical mastitis according to Kelly [38].

### Samples

A total of 336 milk samples collected from 177 dairy goats (mastectomy was recorded in six halves, and complete atrophy in one half was recorded in 12 animals) were employed in this study. 15 ml of milk was collected in a sterile tube under strict hygienic measures from each half after disinfection of the teat with 70% alcohol. The first three squirts from each half were discarded. Milk samples were kept on ice and transferred immediately to the laboratory for the assessment of SCC and bacteriological examination within 24 h.

### SCC

Milk SCC was assessed in 180 apparently normal half milk samples by The NucleoCounter^®^ SCC instrument that is based on ChemoMetec’s proven technology of Fluorescence image cytometry. This method uses the single-use SCC-Cassette™ sampling and measuring device, the NucleoCounter^®^ SCC-100™ system. The measurement range of the NucleoCounter^®^ SCC-100™ is between 1×10^4^ cells/ml and 2×10^6^ cells/ml.

### Bacteriological examination

Bacteriological examination of milk samples was performed according to Sztachańska *et al*. [39]. Briefly, 10 μl of milk were cultivated on Blood Agar Base (BioMérieux Poland), MacConkey Agar (BTL, Poland), Mannitol salt agar (Oxoid Ltd., England), and Edwards Medium (Oxoid Ltd., England). Plates were incubated at 37°C and read at 24 and 48 h later. Colonies were identified by their colony morphology and Gram staining. Detailed identification of isolated bacteria was performed using standard biochemical tests and API-Coryne system (bioMérieux Poland).

For *C. pseudotuberculosis* diagnosis, milk samples were inoculated onto brain heart infusion (BHI) agar supplemented with 5% defibrinated sheep blood and chocolate agar. The plates were incubated aerobically for approximately 48 h at 37°C. Colonies that morphologically resembled *C. pseudotuberculosis* were Gram stained. Gram-positive colonies were further tested for urease activity, synergistic hemolytic activity with Christie, Atkins, and Munch-Peterson factor from *Rhodococcus equi* and carbohydrate fermentation (glucose, lactose, and sucrose). Strains that were positive for urease and glucose fermentation and negative for lactose and sucrose fermentation were identified as *C. pseudotuberculosis* [40].

### Molecular diagnosis of C. pseudotuberculosis [28]

#### Extraction of DNA

According to the above-mentioned bacteriological isolation and identification, *C. pseudotuberculosis* colonies were grown in BHI broth (BHI; Oxoid) at 37°C for 48–72 h before DNA extraction. Bacterial DNA was extracted using QIAamp DNA Mini Kit (Catalogue no. 51304) according to the prescribed instructions.

#### Primers, amplification conditions, and agarose gel electrophoresis

The oligonucleotide primers used in this study are listed in Table-1. Primers targeting the *Pld* and *rpoB* genes of *C. pseudotuberculosis* were obtained from previously published work [41,42].

**Table-1:** List of oligonucleotide primers used in this study and their references.

Gene	Primers	Sequence (5′→3′)	PCR product	References
*Pld*	*PLD-F*	ATAAGCGTAAGCAGGGAGCA	203 bp	[41]
	*PLD-R2*	ATCAGCGGTGATTGTCTTCCAGG		
*rpoB*	*C2700F*	CGWATGAACATYGGBCAGGT	406 bp	[42]
	*C3130R*	TCCATYTCRCCRAARCGCTG		

*Pld*=Phospholipase D, PCR=Polymerase chain reaction, *rpoB*=β-subunit of RNA polymerase

Amplification reaction mixtures were prepared in volumes of 50 μL containing 5 μL of 10× PCR master mix (Fermentas, Vilnius, Lithuania), 5 μl of 25 mM MgCl2, 0.2 μL of 10 mM dNTP mixture (Fermentas), 2 U of Taq DNA polymerase (Fermentas), 1 μmol of 25 mM each primer, and 5 μL of template. PCR was performed in a DNA thermocycler (Thermo Electron Corp., Waltham, MA, USA) and amplifications were performed using protocols listed in Table-2. The negative control contained sterile, DNase/RNase free, and DEPC (diethylpyrocarbonate)-treated water (Applichem) instead of DNA template. As a positive control, DNA isolated from *C. pseudotuberculosis* Pl 18 strain (isolated strain from a sheep with CLA). The amplified products were analyzed by electrophoresis on a 2% (w/v) agarose gel against gel pilot 100 bp ladder (Qiagen, USA, Cat. No. 239035). Amplified products were visualized using a gel documentation system, and the data were analyzed through computer software. PCR products with a molecular size of 203 bp (*Pld*) and 406 bp (*rpoB*) were considered positive for *C. pseudotuberculosis*.

**Table-2:** Cycling conditions of the different primers during PCR.

Gene	Primary denaturation	Secondary denaturation	Annealing	Extension	Number of cycles	Final extension
*PLD*	94°C 5 min	94°C 30_s_	56°C 30_s_	72°C 30_s_	35	72°C 10 min
*rpoB*	94°C 5 min	94°C 30_s_	52°C 45_s_	72°C 45_s_	35	72°C 10 min

*Pld*=Phospholipase D, PCR=Polymerase chain reaction, *rpoB*=β-subunit of RNA polymerase

## Results

Clinical examination of 177 dairy goats revealed that the presence of symptoms suggestive for clinical mastitis in 54 animals (30.5%) and 123 animals (69.5%) was apparently healthy with normal milk secretion (Table-3). Animals were diagnosed for clinical mastitis if suffer from pain on milking, swelling of udder, hardness, and necrosis in udder, decreased milk production, or changes in milk.

**Table-3:** Results of clinical examination of 177 dairy goats.

Health status	Number of animals (%)
Clinical mastitis	54 (30.5)
Clinically healthy	123 (69.5)
Total	177 (100)

Bacteriological examination of 336 milk samples revealed that single infection in 147 milk samples (43.75%), mixed infection in 84 milk samples (25%), and 105 milk samples (31.25%) did not show any microbial growth on the utilized media (Table-4). A total number of 315 bacterial isolates were recovered. The most predominant bacterial study was CNS (41.96%) and *S. aureus* (37. 5%). *C. pseudotuberculosis* was isolated and identified from 24 milk samples (7.14%) (Table-5).

**Table-4:** Results of bacteriological examination of 336 quarter milk samples of 68 lactating cows.

Bacteriological status	Number of samples (%)
Negative samples	105 (31.25)
Single pathogen	147 (43.75)
Mixed infection	84 (25)
Total	336 (100)

**Table-5:** The identified pathogens with their prevalence rate in half milk samples.

Identified bacteria	Number of samples (%)
*C. pseudotuberculosis*	24 (7.14)
CNS	141 (41.96)
*S. aureus*	126 (37.5))
*E. coli*	15 (4.46)
*Streptococci*	9 (2.68)
Total	315

*C. pseudotuberculosis*=*Corynebacterium pseudotuberculosis,* CNS=*Coagulase-negative staphylococci, S. aureus*=*Staphylococcus aureus, E. coli*=*Escherichia coli*

For SCC assessment, 96 milk samples (53.33%) had SC ≥1,000,000 cells/ml and represent SCM, and 84 (46.67%) milk samples had SCC ≤1,000,000 cells/ml (Table-6).

**Table-6:** Results of SCC estimation in 180 apparently healthy half milk samples.

SCC	n (%)
SCC≥1,000,000	96 (51.67)
SCC≤1,000,000	84 (46.67)

SCC=Somatic cell count

**Figure-2:**
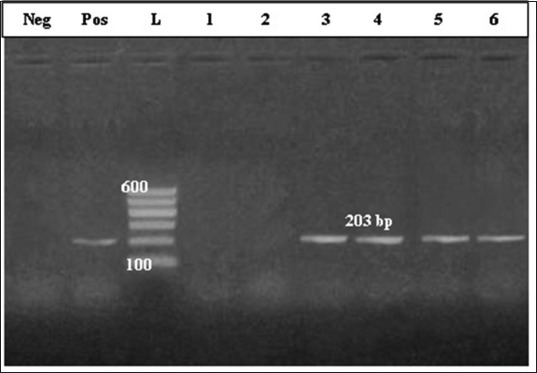
Polymerase chain reaction-amplified DNA fragment of 203 bp and specific for the phospholipase D gene of *Corynebacterium pseudotuberculosis*. Lane 1: Control negative; Lane 2: Control positive; Lane 3: Molecular marker; Lanes 4-9 culture-positive samples.

**Figure-3:**
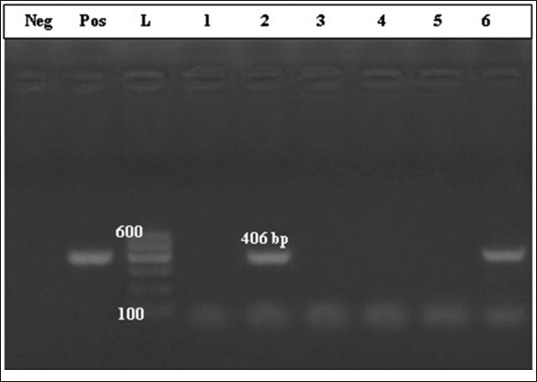
Polymerase chain reaction-amplified DNA fragment of 406 bp and specific for the β-subunit of RNA polymerase gene of *Corynebacterium pseudotuberculosis*. Lane 1: Control negative; Lane 2: Control positive; Lane 3: Molecular marker; Lanes 4-9 culture-positive samples.

Molecular detection of *C. pseudotuberculosis* virulence genes revealed that PCR-amplified DNA fragment of 203 bp and specific for the *Pld* gene of *C. pseudotuberculosis* was evidenced in 16 samples of 24 bacteriologically diagnosed isolates as *C. pseudotuberculosis* (66.66%) (Figure-2). While PCR amplified DNA fragment of 406 bp, and specific for the *rpo*B gene was evidenced in 6 samples of 24 bacteriologically diagnosed isolates as *C. pseudotuberculosi*s (25%) (Figure-3).

Mastitis continues to be an important threat confronting the goat milk industry, particularly in developing countries where the goat milk production has an important socioeconomic role.

In this study, clinical examination of the udder of 177 dairy goats revealed that 54 animals (30.5%) demonstrated clinical mastitis according to Blood and Radostits [43], and 123 animals (69.5%) were clinically healthy and secreted milk was normal (Table-3).

Clinical mastitis is easy to be detected, while SCM is often difficult to recognize due to a lack of reliable diagnostic methods, particularly at the farm level. Herein, SCM was diagnosed by SCC estimation in milk secretion of 180 apparently healthy udder halves. Its incidence was 53.33%.

The observed decreased milk yield during IMI was explained by Petersson-Wolfe *et al*. [44] that an influx of neutrophils will pass between milk-producing cells of mammary gland and into the alveolar lumen resulting in damage of milk-secreting cells.

The prevalence of SCM in dairy goats was estimated in previous studies to be 5-30% or even higher, with about 6 times the incidence of clinical affections [10]. Others reported that the proportion of SCM in udder halves was 35-70% [45]. In Brazil, the prevalence of mastitis in dairy goats was about 75%, and most of infections were subclinical [46].

In a recent study carried out in China, SCM was diagnosed in 45.82% of examined dairy goats [47], while it was 18% in Sweden [13] and 30.2% in India [48]. The authors attributed this high prevalence of SCM to be attributed to poor milking hygiene and the less awareness of SCM impact. Poor management represented by allowing infected animals to be in contact with healthy ones.

Our results concerning bacteriological findings proved single infection in 147 milk samples (43.75%) and mixed infection in 84 milk samples (25%); while 105 (31.25%) milk samples were negative (Table-4), and the identified pathogens were CNS (41.96%) and *S. aureu*s (37.5%). *C. pseudotuberculosis* was isolated and identified from 33 milk samples (7.14%) (Table-5).

These results are too great extent in accord with previous studies, where staphylococci were recorded to be the most prevalent bacteria in cases of mastitis and account for 90% of isolated bacteria [10,49-51]. Furthermore, CNS recorded to have the capability of increasing SCC in goat milk and occur at over 50% in most studies of goat SCM [47]. About streptococci, it was reported to be the major pathogens for their severe inflammation, but they are less common in SCM in goats [47].

In 2015, a similar study carried on dairy goats and revealed that the incidence of IMI with CNS, *S. aureus*, *Escherichia coli*, and Streptococcus spp. was 59.52%, 15.24%, 11.43%, and 10.95%, respectively. The study concluded that CNS were the predominant pathogens [47]. Furthermore, Contreras *et al*. [52] recorded that CNS were the most predominant causative agent of mastitis in does. Another research group reported that CNS were the most predominant bacteria and encountered for 81.5% of milk samples from SCM infected does [53].

However, CNS are less pathogenic than *S. aureus*, it induces persistent SCM with markedly high SCC [52]. *C. pseudotuberculosis* was isolated and identified in 24 half milk samples (7.14%), all of these samples had mixed infection mostly with CNS. Molecular diagnosis indicated that 16 isolates harbor gene sequence specific for *Pld* gene (Figure-2), while gene sequence specific for the *rpoB* gene was diagnosed in six isolates only (Figure-3).

*C. pseudotuberculosis* infection results in acute suppurative mastitis or chronic encapsulated abscesses in the udder [54] causing economic losses due to decreased milk production, reproductive inefficiency, carcass condemnation, and rare cases of death [17]. *C. pseudotuberculosis* has been reported to have public health significance, causing lymphadenitis [55].

Once infection occurs in animal, the enlarged lymph nodes and abscesses can rupture and contaminate the milk, lambs, kids, other animals, and environment [17,56]. In the previous study, the prevalence of *Corynebacterium* sp. was 4.13% in dairy goats, mostly in association with *E. coli* [14]. This is in accordance with the results reported by McDougal *et al*. [57], who identify them as part of the microbial agents of mastitis in goats.

The identification of virulence factors involved in bacterial pathogenicity in mammary gland is essential for the development of effective control and prevention of SCM in goats and acts as ideal targets for accurate detection and identification.

To date, in *C. pseudotuberculosis*, the most important identified virulence determinant is *Pld* [58]. *Pld* increases vascular permeability *in vivo*. It has dermonecrotic properties and reduces the viability of neutrophils [59]. Studies with *C. pseudotuberculosis* strains with inactivated *Pld* demonstrated the necessity of *Pld* for CLA establishment [24,60]. Mutant strains were found to be unable to cause abscessation of the lymph nodes. Additional evidence for the importance of *Pld* *in* *vivo* comes from the observation that vaccination with formulations in which *Pld* is the major component protects against subsequent disease challenge [61].

## Conclusion

*C. pseudotuberculosis* must be considered for routine bacteriological examination of milk from dairy goats, particularly herds with a history of CLA. *Pld* gene-based PCR is more reliable than *rpoB* gene-based ones for the diagnosis of *C. pseudotuberculosis*.

## Authors’ Contributions

AMN, HAH, and AMG designed and planned for this study. HAH and KAA performed the fieldwork and collected the samples. AMN and SAE were responsible for bacteriological examination and SCC. HAH and AMG interpreted the results and reviewed the article. HAH drafted the manuscript. All authors read and approved the final manuscript.

## References

[1] FAO (2013). Food and Agriculture Organization of the United Nations statistical Databases.

[2] Skapetas B, Bampidis V (2016). Goat production in the world: Present situation and trends. Livest. Res. Rural Dev.

[3] Zenebe T, Ahmed N, Kabeta T, Kebede G (2014). Review on medicinal and nutritional values of goat milk. Acad. J. Nutr.

[4] Barrón-Bravo OG, Gutiérrez-Chávez AJ, Ángel-Sahagún CA, Montaldo HH, Shepard L, Valencia-Posadas M (2013). Losses in milk yield, fat and protein contents according to different levels of somatic cell count in dairy goats. Small Rumin. Res.

[5] Jimenez-Granado R, Sanchez-Rodriguez M, Arce C, Rodriguez-Estevez V (2014). Factors affecting somatic cell count in dairy goats: A review. Span. J. Agric. Res.

[6] Ceniti C, Britti D, Santoro AML, Musarella R, Ciambrone L, Casalinuovo F, Costanzo N (2017). Phenotypic antimicrobial resistance profile of isolates causing clinical mastitis in dairy animals. Ital. J. Food Saf.

[7] Gould LH, Mungai E, Behravesh CB (2014). Outbreaks attributed to cheese: Differences between outbreaks caused by unpasteurized and pasteurized dairy products, United States, 1998–2011. Foodborne Pathog. Dis.

[8] Contreras A, Sierra D, Sánchez A, Corrales JC, Marco JC, Paape MJ, Gonzalo C (2007). Mastitis in small ruminants. Small Rumin. Res.

[9] Koop G, van Werven T, Schuilling HJ, Nielen M (2010). The effect of subclinical mastitis on milk yield in dairy goats. J. Dairy Sci.

[10] Gelasakis AI, Angelidis AS, Giannakou R, Filioussis G, Kalamaki MS, Arsenos G (2016). Bacterial subclinical mastitis and its effect on milk yield in low-input dairy goat herds. J. Dairy Sci.

[11] Haenlein G.F.W (2002). Relationship of somatic cell counts in goat milk to mastitis and productivity. Small Rumin. Res.

[12] Silanikove N, Merin U, Shapiro F, Leitner G (2014). Subclinical mastitis in goats is associated with upregulation of nitric oxide-derived oxidative stress that causes reduction of milk antioxidative properties and impairment of its quality. J. Dairy Sci.

[13] Persson Y, Olofsson I (2011). Direct and indirect measurement of somatic cell count as indicator of intramammary infection in dairy goats. Acta Vet. Scand.

[14] Hristov K, Popova T, Pepovich R, Nikolov B (2016). Characterization of microbial causative agents of subclinical mastitis in goats in Bulgaria. Int. J. Curr. Microbiol. App. Sci.

[15] Shpigel NY, Elad D, Yeruham I, Winkler M, Saran A (1993). An outbreak of *Corynebacterium pseudotuberculosis* infection in an Israeli dairy herd. Vet. Rec.

[16] Yeruham I, Braverman Y, Shpigel NY, Chizov-Ginzburg A, Saran A, Winkler M (1996). Mastitis in dairy cattle caused by *Corynebacterium pseudotuberculosis* and the feasibility of transmission by houseflies. Vet. Q.

[17] Brown CC, Olander HJ (1987). Caseous lymphadenitis of goats and sheep: A review. Vet. Bull.

[18] Jung BY, Lee SH, Kim HY, Byun JW, Shin DH, Kim D, Kwak D (2015). Serology and clinical relevance of *Corynebacterium pseudotuberculosis* in native Korean goats (*Capra hircus coreanae*). Trop. Anim. Health Prod.

[19] Guimarães ADS, Borges F, Pauletti RB, Seyffert N, Ribeiro D, Lage AP, Heinemann MB, Miyoshi A, Maria A, Gouveia G, Federal U, Gerais DM, Av U, Carlos A, Postal C, Cep U, Horizonte B, Gerais M (2011). *Caseous lymphadenities*: Epidimology, diagnosis and control. IIOAB J.

[20] Arsenault J, Girard C, Dubreuil P, Daignault D, Galarneau JR, Boisclair J, Simard C, Bélanger D (2003). Prevalence of and carcass condemnation from maedi-visna, paratuberculosis and caseous lymphadenitis in culled sheep from Quebec, Canada. Prev. Vet. Med.

[21] Dorella FA, Pacheco LGC, Oliveira SC, Miyoshi A, Azevedo V (2006). *Corynebacterium pseudotuberculosis*: Microbiology, biochemical properties pathogenesis and molecular studies of virulence. Vet. Res.

[22] Hemond V, Rosenstingl S, Auriault ML, Galanti MJ, Gatfosse M (2009). Axillary lymphadenitis due to *Corynebacterium pseudotuberculosis* in a 63-year-old patient. Med. Mal. Infect.

[23] Boschert V, Berger A, Konrad R, Huber I, Hörmansdorfer S, Zöls S, Eddicks M, Ritzmann M, Sing A (2014). *Corynebacterium* species nasal carriage in pigs and their farmers in Bavaria, Germany: Implications for public health. Vet. Rec.

[24] Hodgson ALM, Carter K, Tachedjian M, Krywult J, Corner LA, McColl M, Cameron A (1999). Efficacy of an ovine caseous lymphadenitis vaccine formulated using a genetically inactive form of the *Corynebacterium pseudotuberculosis* phospholipase D. Vaccine.

[25] Baird GJ, Fontaine MC (2007). *Corynebacterium pseudotuberculosis* and its role in ovine caseous lymphadenitis. J. Comp. Pathol.

[26] Corrêa JI, Stocker A, Trindade SC, Vale V, Brito T, Bastos B, Raynal JT, Miranda PM, Alcantara AC, Freire SM, Costa LM, Meyer R (2018). *In vivo* and *in vitro* expression of five genes involved in *Corynebacterium pseudotuberculosis* virulence. AMB Expr.

[27] Khamis A, Raoult D, La Scola B (2004). rpoB gene sequencing for identification of *Corynebacterium* species. J. Clin. Microbiol.

[28] Pacheco LGC, Pena RR, Castro TLP, Dorella FA, Bahia RC, Carminati R, Frota MNL, Oliveira SC, Meyer R, Alves FSF, Miyoshi A, Azevedo V (2007). Multiplex PCR assay for identification of *Corynebacterium pseudotuberculosis* from pure cultures and for rapid detection of this pathogen in clinical samples. J. Med. Microbiol.

[29] Britten AM (2012). The role of diagnostic microbiology in mastitis control programs. Vet. Clin. N. Am. Food Anim. Pract.

[30] Ashraf A, Imran M (2018). Diagnosis of bovine mastitis: From laboratory to farm. Trop. Anim. Health Prod.

[31] Jashari R, Piepers S, De Vliegher S (2016). Evaluation of the composite milk somatic cell count as a predictor of intramammary infection in dairy cattle. J. Dairy Sci.

[32] Schukken YH, Wilson DJ, Welcome F, Garrison-Tinofsky L, Gonzales RN (2003). Monitoring udder health and milk quality using somatic cell counts. Vet. Res.

[33] Paape MJ, Capuco AV (1997). Cellular defense mechanisms in the udder and lactation of goats. J. Anim. Sci.

[34] Schaeren W, Maurer J (2006). Prevalence of subclinical udder infections and individual somatic cell counts in three dairy goat herds during a full lactation. Schweiz Arch. Tierheilkd.

[35] Cantekin Z, Ergün Y, Doǧruer G, Saribay MK, Solmaz H (2015). Comparison of PCR and culture methods for diagnosis of subclinical mastitis in dairy cattle. Kafkas Univ. Vet. Fak. Derg.

[36] Charaya G, Sharma A, Kumar A, Goel P, Singh M (2015). Detection of major mastitis pathogens by multiplex polymerase chain reaction assay in buffalo milk. Indian J. Anim. Sci.

[37] Ashraf A, Imran M, Yaqub T, Tayyab M, Shehzad W, Thomson PC (2017). A novel multiplex PCR assay for simultaneous detection of nine clinically significant bacterial pathogens associated with bovine mastitis. Mol. Cell. Probe.

[38] Kelly WG (1984). Veterinary Clinical Diagnosis.

[39] Sztachańska M, Barański W, Janowski T, Pogorzelska J, Zduńczyk S (2016). Prevalence and etiological agents of subclinical mastitis at the end of lactation in nine dairy herds in North-East Poland. Pol. J. Vet. Sci.

[40] Rebouças MF, Portela RW, Lima DD, Loureiro D, Bastos BL, Moura-Costa LF, Vale VL, Miyoshi A, Azevedo V, Meyer R (2011). *Corynebacterium pseudotuberculosis* secreted antigen-induced specific gamma-interferon production by peripheral blood leukocytes: Potential diagnostic marker for caseous lymphadenitis in sheep and goats. J. Vet. Diagn. Invest.

[41] Ilhan Z (2013). Detection of *Corynebacterium pseudotuberculosis* from sheep lymph nodes by PCR. Revue. Méd. Vét.

[42] Sammra O, Balbutskaya A, Hijazin M, Nagib S, Alber J, Lämmler C, Abdulmawjood A, Prenger-Berninghoff E, Timke M, Kostrzewa M, Siebert U (2014). Further studies on *Arcanobacterium phocisimile*: A novel species of genus *Arcanobacterium*. J. Vet. Med.

[43] Blood DC, Radostits OM (1989). Veterinary Medicine.

[44] Petersson-Wolfe CS, Tholen AR, Currin J, Leslie KE (2013). Practical methods for mastitis control. WCDS Adv. Dairy Technol.

[45] Menzies PI, Ramanoon SZ (2001). Mastitis of sheep and goats. Vet. Clin. N. Am. Food Anim. Pract.

[46] Peixoto RM, Mota RA, Costa MM (2010). Small ruminant mastitis in Brazil. Pesq. Vet. Bras.

[47] Zhao Y, Liu H, Zhao X, Gao Y, Zhang M, Chen D (2015). Prevalence and pathogens of subclinical mastitis in dairy goats in China. Trop. Anim. Health Prod.

[48] Sreeja S, Bineesh PP, Vijayakumar K, Saseendranath MR (2013). Evaluation of California mastitis test (CMT) as a screening method for subclinical mastitis in Malabari goats. Indian J. Anim. Res.

[49] Koop G, De Visscher A, Collar CA, Bacon DA, Maga EA, Murray JD, Supré K, De Vliegher S, Haesebrouck F, Rowe JD, Nielen M, van Werven T (2012). Short communication: Identification of coagulase-negative Staphylococcus species from goat milk with the API Staph identification test and with transfer RNA-intergenic spacer PCR combined with capillary electrophoresis. J. Dairy Sci.

[50] Marogna G, Pilo C, Vidili A, Tola S, Schianchi G, Leori SG (2012). Comparison of clinical findings, microbiological results, and farming parameters in goat herds affected by recurrent infectious mastitis. Small Rumin. Res.

[51] McDougall S, Malcolm D, Prosser C.G (2014). Prevalence and incidence of intramammary infections in lactating dairy goats. N. Z. Vet. J.

[52] Contreras A, Paape MJ, Di Carlo AL, Miller RB, Rainard P (1997). Evaluation of selected antibiotic residue screening tests for milk from individual goats. J. Dairy Sci.

[53] Salaberry SR, Saidenberg AB, Zuniga E, Melville PA, Santos FG, Guimarães EC, Gregori F, Benites N.R (2015). Virulence factors genes of *Staphylococcus* spp. Isolated from caprine subclinical mastitis. Microb. Pathog.

[54] Valli V.E.O, Parry BW, Jubb KVF, Kennedy PC, Palmer N (1993). Caseous lymphadenitis. Pathology of Domestic Animals.

[55] Peel MM, Palmer GG, Stacpoole AM, Kerr TG (1997). Human lymphadenitis due to *Corynebacterium pseudotuberculosis*: Report of ten cases from Australia and review. Clin. Infect. Dis.

[56] Stoops SG, Renshaw HW, Thilsted JP (1984). Ovine caseous lymphadenitis: Disease prevalence, lesion distribution, and thoracic manifestations in a population of mature culled sheep from Western United States. Am. J. Vet. Res.

[57] McDougall S, Pankey W, Delaney C, Barlow J, Murdough PA, Scruton D (2002). Prevalence and incidence of subclinical mastitis in goats and dairy ewes in Vermont, USA. Small Rumin. Res.

[58] Hodgson AL, Bird P, Nisbet IT (1990). Cloning, nucleotide sequence, and expression in *Escherichia coli* of the phospholipase D gene from *Corynebacterium pseudotuberculosis*. J. Bacteriol.

[59] Yozwiak ML, Songer JG (1993). Effect of *Corynebacterium pseudotuberculosis* phospholipase D on viability and chemotactic responses of ovine neutrophils. Am. J. Vet. Res.

[60] Simmons CP, Dunstan SJ, Tachedjian M, Krywult J, Hodgson AL, Strugnell RA (1998). Vaccine potential of attenuated mutants of *Corynebacterium pseudotuberculosis* in sheep. Infect. Immun.

[61] Eggleton DG, Middleton HD, Doidge CV, Minty DW (1991). Immunisation against ovine caseous lymphadenitis: Comparison of *Corynebacterium pseudotuberculosis* vaccines with and without bacterial cells. Aust. Vet. J.

